# High-Quality Complete Genome Sequences of Three Pseudomonas aeruginosa Isolates Retrieved from Patients Hospitalized in Intensive Care Units

**DOI:** 10.1128/MRA.01624-18

**Published:** 2019-02-28

**Authors:** Bárbara Magalhães, Laurence Senn, Dominique S. Blanc

**Affiliations:** a Service of Hospital Preventive Medicine, Lausanne University Hospital, Lausanne, Switzerland; University of Maryland School of Medicine

## Abstract

Pseudomonas aeruginosa is one of the major Gram-negative pathogens responsible for hospital-acquired infections. Here, we present high-quality genome sequences of isolates from three P. aeruginosa genotypes retrieved from patients hospitalized in intensive care units. PacBio reads were assembled into a single contig, which was afterward corrected using Illumina HiSeq reads.

## Abstract (precis)

Pseudomonas aeruginosa is one of the major Gram-negative pathogens responsible for hospital-acquired infections. Here, we present high-quality genome sequences of isolates from three P. aeruginosa genotypes retrieved from patients hospitalized in intensive care units.

## ANNOUNCEMENT

Pseudomonas aeruginosa is an opportunistic Gram-negative pathogen which is identified as one of the most frequent microorganisms in intensive care units (ICUs) (1, 2). Following an unexplained increase in P. aeruginosa incidence in the ICUs of the University Hospital of Lausanne, all clinical and environmental isolates from 2010 to 2014 were typed. Most patients harbored isolates from three sequence types (STs), ST1076, ST253, and ST17. To further investigate the epidemiology of this pathogen in the ICUs with short-read whole-genome sequencing, a complete reference genome was constructed for each ST. The first clinical isolate collected from each of the three STs was selected for that purpose, H25883 (ST1076), H26023 (ST253), and H26027 (ST17).

Single colonies were inoculated in 5 ml of lysogeny broth (LB) and incubated for 4 h to reach early exponential phase. Extraction of the genomic DNA was performed on 1.5-ml cultures using the GenElute bacterial genomic DNA kit (Sigma-Aldrich, St. Louis, MO, USA). The genomic DNA (gDNA) was subsequently used for library preparation according to the PacBio standard protocol with the BluePippin size selection system (Sage Science). The finished libraries were sequenced on a PacBio RS II instrument using P6-C4 chemistry, for 360-min movies, and yielded 100,236 to 103,875 reads with an average size of 19,375 to 19,604 bp. Hierarchical Genome Assembly Process (HGAP3) version 2.3.0 (3) from the SMRT Analysis software suite (PacBio) was used to assemble the PacBio reads with a minimum seed read length of 6 kb. All genomes were manually circularized using the Minimus pipeline (4) included in Amos (5), merging the overlapping extremities of the main contig. A single circular contig was produced for isolates H25883, H26023, and H26027, with the following genome sizes and coverages: 6,706,793 bp and 223× for H25883, 6,729,215 bp and 217× for H26023, and 7,079,586 bp and 228× for H26027.

The extracted gDNA was also used for library preparation with the Nextera DNA library preparation kit (Illumina, Inc., San Diego, CA, USA) for 100-bp paired-end sequencing on an Illumina HiSeq 2500 platform, aiming for 100-fold coverage. Illumina HiSeq reads were mapped against the assembled PacBio contigs with BWA-MEM, and single nucleotide polymorphisms (SNPs) and indels were identified and corrected using Pilon version 1.22 (6), with a minimum size for unclosed gaps of 10. The genotypes, final genome sizes, and G+C contents of the three final corrected circular genomes are represented in Table 1.

**TABLE 1:** Metadata of the three complete corrected genomes of each genotype

Isolate no.	Sequence type	*N*_50_ read length (bp)	GenBank accession no.	SRA accession no. by read type		Genome size (bp)	G+C content (%)	No. of CDSs
				Illumina	PacBio			
H25883	1076	26,667	CP033686	SRX5329115	SRX5322128	6,706,800	66.15	6,216
H26023	253	26,676	CP033685	SRX5329116	SRX5322127	6,729,216	66.21	6,246
H26027	17	27,385	CP033684	SRX5329117	SRX5322129	7,079,598	66.07	6,629

A total of 6,400 to 6,806 genes were predicted with Prokaryotic Genome Annotation Pipeline (PGAP) (7) and 6,216 to 6,629 coding sequences (CDSs) annotated, together with 63 to 64 tRNAs and 4 rRNA operons.

### Data availability.

The complete genome sequences for the three Pseudomonas aeruginosa isolates have been deposited in DDBJ/ENA/NCBI, and the PacBio and Illumina reads are available in the NCBI Sequence Read Archive. The respective accession numbers are listed in Table 1.
